# Ampicillinase C Beta-lactamase Producers among Isolates of Enterobacteriaceae in a Tertiary Care Centre: A Descriptive Cross-sectional Study

**DOI:** 10.31729/jnma.7536

**Published:** 2022-08-31

**Authors:** Shusila Khadka, Achut Barakoti, Ram Prasad Adhikari, Laxmi Kant Khanal, Jyotshna Sapkota

**Affiliations:** 1 Department of Microbiology, Nepal Medical College and Teaching Hospital, Jorpati, Kathmandu Nepal

**Keywords:** *antibiotic*, *beta-lactamase*, *Enterobacteriaceae*, *gram-negative bacteria*

## Abstract

**Introduction:**

Ampicillinase C beta-lactamase-producing organisms are often resistant to multiple antimicrobial agents, and therapeutic options against these pathogens are limited. Limited information is available regarding Ampicillinase C beta-lactamase producers. The aim of this study was to find out the prevalence of Ampicillinase C beta-lactamase producers among isolates of *Enterobacteriaceae* in a tertiary care centre.

**Methods:**

A descriptive cross-sectional study was carried out in the Clinical Microbiology Laboratory of a tertiary care centre from May 2021 to October 2021. Ethical approval was received from the Institutional Review Committee (Reference number: 044-077/078). Isolates of *Enterobacteriaceae* from various clinical samples were collected by convenience sampling. Ampicillinase C screening for beta-lactamase producers among the *Enterobacteriaceae* isolates was done using cefoxitin (30 μg) disc. Detection of Ampicillinase C beta-lactamase producers among the screen-positive isolates was done by cefoxitin-cloxacillin double-disc synergy test. An increase in the zone size of ≥4 mm was considered as Ampicillinase C beta-lactamase producers. Point estimate and 95% Confidence Interval were calculated.

**Results:**

Among the total 481 isolates of *Enterobacteriaceae,* 49 (10.19%) (7.50-12.90, 95 % Confidence Interval) were detected as Ampicillinase C beta-lactamase producers among isolates of *Enterobacteriaceae* .

**Conclusions:**

The prevalence of Ampicillinase C beta-lactamase producers was lower than in other studies done in similar settings. Meropenem could be a drug of choice for the treatment of infections due to Ampicillinase C beta-lactamase-producing gram-negative bacteria.

## INTRODUCTION

*Enterobacteriaceae,* a large diverse group of facultative Gram-negative rods, are the common causative agents of various infections and are associated with resistance to multiple antibiotics.^1^ Ampicillinase C beta-lactamase (AmpC β-lactamase) has become a major cause of resistance to cephalosporins and other beta-lactam antibiotics.^2^

AmpC β-lactamase producers are resistant to cephalosporins, beta-lactamase inhibitors and to other commonly used antibiotics.^13^ They have high potential to transfer the drug resistance to other bacteria horizontally. Also, they may appear susceptible to expanded-spectrum cephalosporins when initially tested when they are actually resistant.^4^ This may lead to inappropriate antimicrobial regimens. So, the detection of AmpC β-lactamase producers is of significant clinical relevance. The cefoxitin-cloxacillin double-disc synergy test has higher sensitivity and specificity for the detection of AmpC β-lactamase producers compared to other phenotypic methods.^5^

Limited information is present in AmpC β-lactamase-producing bacteria.

The objective of this study was to find out the prevalence of AmpC β-lactamase producers among Enterobacteriaceae isolates in a tertiary care centre.

## METHODS

This was a descriptive cross-sectional study carried out among isolates of *Enterobacteriaceae* in the Clinical Microbiology Laboratory of Nepal Medical College and Teaching Hospital from May 2021 to October 2021. Ethical approval was received from the Institutional Review Committee (Reference number: 044-077/078). All the clinical isolates of *Enterobacteriaceae* were included whereas duplicated samples/isolates were excluded from the study. Convenience sampling was done and the sample size was calculated using the formula:


$$\begin{array}{ll} \text{n} & ={\text{Z}}^{2}\times \frac{\text{p}\times \text{q}}{{\text{e}}^{\text{2}}}\\ & =1.96^{2}\times \frac{0.407\times 0.593}{0.05^{2}}\\ & =371 \end{array}$$

Where,

n= minimum required sample size
Z= 1.96 at 95% Confidence Interval (CI)
p= prevalence of AmpC β-lactamase producers, 40.7%^6^
q= 1-p
e= margin of error, 5%

A total of 481 isolates of *Enterobacteriaceae* were taken. All clinical samples (pus, blood, urine, stool, sputum, pleural fluid, CSF) received for culture and sensitivity in the Clinical Microbiology laboratory were processed following standard protocol.^7^ In brief, the specimens were inoculated in culture plates [urine in cysteine lactose electrolyte deficient (CLED) media], pus in blood agar and Mac-Conkey agar, sputum and body fluids in blood agar, Mac-Conkey agar and chocolate agar. All inoculated plates were incubated at 37°C for 24 hours aerobically. All received blood culture bottles were incubated at 37°C and after 24 hours, sub-cultured in blood agar and Mac Conkey agar every alternate day for seven days. Bacterial isolates of the family Enterobacteriaceae were then identified further by studying colony characters, gram stain and biochemical tests. Antibiotic susceptibility test was done by Kirby Bauer disc diffusion method following standard clinical laboratory and standard institute (CLSI) guidelines.^8^ Screening of AmpC β-lactamase producers was done by using a cefoxitin disc (30 μg). Isolates were considered potential AmpC β-lactamase producers (screen positive) if the zone of inhibition for cefoxitin was ≤18 mm (CLSI susceptible breakpoint).^9-11^

Isolates of *Enterobacteriaceae* with screening test positive were subjected to cefoxitin-cloxacillin (30-200 μ) double-disk synergy test. A difference in the cefoxitin-cloxacillin inhibition zones minus the cefoxitin-alone zones of 4 mm or more was considered indicative of AmpC β-lactamase producers.^5,9,12^

Data were entered and analysed in Microsoft Excel 2013. Point estimate and 95% Confidence Interval were calculated.

## RESULTS

Among a total of 481 isolates of *Enterobacteriaceae,* 49 (10.19%) (7.50-12.90, 95% CI) were AmpC β-lactamase producers (Table 1).

**Table 1:** Distribution of the isolates of Enterobacteriaceae among the AmpC β-lactamase producers (n= 49).

Producers Isolate	AmpC β-lactamase producers n (%)
Escherichia coli	38 (77.55)
Klebsiella pneumoniae	11 (22.45)

AmpC β-lactamase producers among Enterobacteriaceae isolates, isfoundtobe higher in urine specimens than lower respiratory specimen (Table 2).

**Table 2:** Proportion of AmpC p-lactamase among Enterobacteriaceae isolates in different clinical specimens (n= 49).

Specimen	Positive AmpC β-lactamase n (%)
Urine	43 (87.75)
Lower respiratory specimens	6 (12.24)

Antibiotic susceptibility testing showed that almost all of the AmpC β-lactamase-producing bacteria were sensitive to carbapenems (meropenem) and tigecycline. On the other hand, they showed marked resistance to fluoroquinolones and cotrimoxazole (Table 3).

**Table 3:** Resistance pattern of AmpC β-lactamase-producing bacteria (n= 49).

Antibiotic	Resistant isolates of *E.coli* (n= 38) n (%)	Resistant isolates of *K. pneumoniae* (n= 11) n (%)
Piperacillin tazobactam	2 (5.26)	4 (36.36)
Ceftriaxone	38 (100)	11 (100)
Ciprofloxacin	26 (68.42)	6 (54.54)
Cotrimoxazole	11 (28.94)	7 (63.63)
Amikacin	2 (5.26)	4 (36.36)
Tigecycline	4 (10.52)	2 (18.18)
Meropenem	-	-

## DISCUSSION

In this study, AmpC β-lactamase detection among *Enterobacteriaceae* isolates was done by a phenotypic method of cefoxitin-cloxacillin double-disc synergy following the screening using cefoxitin disc. Among the 481 isolates of *Enterobacteriaceae,* 16.83% were positive for AmpC β-lactamase screening test out of which 49 (10.2%) isolates were AmpC β-lactamase producers. *Enterobacteriaceae* are responsible for a large proportion of serious, life-threatening infections and resistance to multiple antibiotics in these organisms is an increasing global public health problem.^13^ The most common resistance of these organisms is to the third generation of cephalosporins. The most common cause of resistance to the third generation of cephalosporins is ESBL producers. But these days, AmpC β-lactamase producers has also contributed to the cause.^4^ *Enterobacteriaceae* producing AmpC β-lactamases have become a major therapeutic challenge these days. The detection of AmpC β-lactamase producers is of significant clinical relevance since AmpC β-lactamase producers may appear susceptible to expanded-spectrum cephalosporins when initially tested when they are not and also have a higher risk of horizontal transfer.^4,14,15^ Cefoxitin resistance without AmpC β-lactamase producers could be due to another mechanism of cefoxitin resistance such as lack of permeation of porins.^16^

In our study, the prevalence of AmpC β-lactamase producers is 10.2% which is lower as compared to a study done in Saudi Arabia and India that shows the prevalence of AmpC β-lactamase among gramnegative isolates at 32.5% and 22.7% respectively.^8,16^ The possible cause of the high rate of detection of AmpC β-lactamase in these studies could be due to the inclusion of all gram-negative bacteria. In a study done in Nepal, 2.7% of the gram-negative bacilli were AmpC β-lactamase producers.^17^ The rate of AmpC β-lactamase producers in this study is lower as compared to ours because variations in the prevalence rate can occur according to time and place. As in our study, this study also showed *E. coli* 71.4% as the predominant AmpC β-lactamase producers. In our study, *E.* coiiaccounted for 77.6% (38 out of 49) and K. pneumonia accounted for 22.4% (11 out of 49) of the AmpC β-lactamase producers. A study done in India also showed *E.coli* as the predominant AmpC β-lactamase producers.^18^

The AmpC β-lactamase producers were susceptible to carbapenems but showed a higher rate of resistance to fluoroquinolones and cotrimoxazole. A study done in Kathmandu, Nepal also showed that AmpC β-lactamase producers exhibited a high rate of resistance to fluoroquinolones and aminoglycosides and susceptibility to carbapenems.^6^ Few other studies also have shown that AmpC β-lactamase producers are susceptible to carbapenems.^16,19^ A study done in Pakistan showed that AmpC β-lactamase-producing E. coli were multidrug-resistant and resistant to cotrimoxazole, ciprofloxacin, and gentamycin.^20^ So, AmpC β-lactamase producers are usually resistant to commonly used antibiotics and carbapenems could be the drug of choice.

In this study, molecular techniques could not be used due to the lack of resources. The study was conducted in a single tertiary care centre.

## CONCLUSIONS

Our study showed that the prevalence of AmpC β-lactamase producers was lower as compared to other studies done in similar settings. In our study, AmpC β-lactamase-producing bacteria were resistant to most of the commonly used antibiotics. Meropenem could be a drug of choice for the treatment of serious infections due to AmpC β-lactamase producing gramnegative bacteria. Identification of AmpC β-lactamase producers may aid in hospital infection control and help the physician to prescribe the most appropriate antibiotic.
